# Validation of PaO_2_:FiO_2_ for predicting hospital mortality in critically ill patients with acute hypoxaemic respiratory failure: a retrospective binational registry-based study

**DOI:** 10.62675/2965-2774.20260221

**Published:** 2026-02-26

**Authors:** Mahesh Ramanan, Benjamin Moran, Ryan Ruiyang Ling, Aidan Burrell, Ashwin Subramaniam, Kollengode Ramanathan, Mallikarjuna Ponnappa Reddy, David Pilcher, Kiran Shekar

**Affiliations:** 1 Metro North Hospital and Health Services Brisbane Queensland Australia Metro North Hospital and Health Services - Brisbane, Queensland, Australia.; 2 Department of Anaesthesia and Department of Intensive Care Gosford Hospital Gosford New South Wales Australia Department of Anaesthesia and Department of Intensive Care, Gosford Hospital -Gosford, New South Wales, Australia.; 3 Yong Loo Lin School of Medicine National University of Singapore National University Health System Singapore Singapore Yong Loo Lin School of Medicine, National University of Singapore, National University Health System - Singapore, Singapore.; 4 Department of Intensive Care Alfred Health Prahran Victoria Australia Department of Intensive Care, Alfred Health - Prahran, Victoria, Australia.; 5 Department of Intensive Care Medicine Dandenong Hospital Monash Health Dandenong Victoria Australia Department of Intensive Care Medicine, Dandenong Hospital, Monash Health - Dandenong, Victoria, Australia.; 6 Thoracic, and Vascular Surgery National University Heart Centre National University Health System Singapore Singapore Cardiothoracic Intensive Care Unit, Department of Cardiac, Thoracic, and Vascular Surgery, National University Heart Centre, National University Health System - Singapore, Singapore.; 7 Department of Anaesthesia and Pain Medicine Nepean Hospital Sydney Australia Department of Anaesthesia and Pain Medicine, Nepean Hospital - Sydney, Australia.; 8 Centre for Outcome and Resource Evaluation Australian and New Zealand Intensive Care Society Melbourne Victoria Australia Centre for Outcome and Resource Evaluation, Australian and New Zealand Intensive Care Society - Melbourne, Victoria, Australia.; 9 Adult Intensive Care Services The Prince Charles Hospital Chermside Queensland Australia Adult Intensive Care Services, The Prince Charles Hospital - Chermside, Queensland, Australia.

**Keywords:** Critical illness, Hospital mortality, Hypoxia, Hypoxaemia, Noninvasive ventilation, Oxygen, Respiratory insufficiency, Registries

## Abstract

**Objective:**

To determine the optimal PaO_2_:FiO_2_ threshold in the first 24 hours of intensive care unit admission, and its associated discriminatory capacity, for prognostication of mortality among critically ill patients.

**Methods:**

This bi-national registry included adult patients admitted to intensive care units in Australia and New Zealand from January-2018 to December-2022. The primary outcome was hospital mortality. Acute hypoxic respiratory failure was defined as PaO_2_:FiO_2_ of < 300 using the worst PaO_2_:FiO_2_ within the first 24 hours of intensive care unit admission. The unadjusted association between PaO_2_:FiO_2_ and hospital mortality was evaluated using restricted cubic splines with four knots to allow for continuous, non-linear associations. To determine the optimal threshold of the PaO_2_:FiO_2_ for predicting hospital mortality, Youden’s method was used to identify the maximum sum of sensitivity and specificity. The area under the receiver operating characteristic curve and Youden’s J-index were calculated to compare pre-specified subgroups.

**Results:**

Among the 662,612 included patients, acute hypoxic respiratory failure was not present in 324,761 (49%) patients, mild in 181,499 (27%) patients, moderate in 128,277 (19%) patients, and severe in 28,125 (4%) patients. The hospital mortality rates, respectively, were 4.9% (15,797/324,761), 7.9% (14,291/181,499), 14% (18,247/128,277), and 31% (8,717/28,125). The association between PaO_2_:FiO_2_ and hospital mortality was non-linear with an inflection point at PaO_2_:FiO_2_ = 200. The area under the ROC curve was 0_._677 (95%CI 0.675 - 0.679) with an optimum PaO_2_:FiO_2_ threshold of 230. (Youden’s J-index of 0.267, sensitivity 56.1% and specificity 70.6%). The area under the ROC curve was 0.627 for patients who required invasive ventilation during their intensive care unit stay, compared with 0.698 for those who did not.

**Conclusion:**

The optimal PaO_2_:FiO_2_ threshold for predicting hospital mortality was 230. PaO_2_:FiO_2_ has low discriminatory capacity in predicting hospital mortality among intensive care unit patients.

Key take-home messages

- The discriminatory capacity for PaO_2_:FiO_2_ measured in the first 24 hours of intensive care admission, to prognosticate mortality for critically ill patients with acute hypoxic respiratory failure, is limited. Hence, PaO_2_:FiO_2_ should be used cautiously for this purpose.
- The inflection point of PaO_2_:FiO_2_ measured in the first 24 hours, below which mortality starts to rise steeply for patients with acute hypoxic respiratory failure is 200.

## INTRODUCTION

Acute hypoxaemic respiratory failure (AHRF) is characterized by acute hypoxaemia, currently defined as a partial pressure of arterial oxygen (PaO_2_) to fraction of inspired oxygen (FiO_2_) ratio (PaO_2_:FiO_2_) less than 300, or oxygen saturation to FiO_2_ ratio less than 315.^(1)^ It may result from respiratory and non-respiratory diseases (cardiovascular or systemic diseases), and can occur in intubated and non-intubated patients. The global burden is substantial, with approximately 1 million patients diagnosed with AHRF each year.^(2-6)^ Acute hypoxaemic respiratory failure is associated with high rates of acute hospitalisation, intensive care unit (ICU) admission, mortality, and morbidity, including physical, psychological, cognitive, and functional morbidity.^(7-9)^

While it is known that mortality rises with a decreasing PaO_2_:FiO_2_, the optimal PaO_2_:FiO_2_ threshold for prognosticating outcomes in patients with critical illness is less well defined. Indeed, multiple scoring systems use a variety of thresholds to define risk.^(10)^ For example, the global definition of acute respiratory distress syndrome (ARDS)^(1)^ uses arbitrary categories of mild, moderate, and severe based on PaO_2_:FiO_2_ cutoffs of 300, 200, and 100, respectively. The discriminatory capacity of PaO_2_:FiO_2_ in identifying patients at higher risk of mortality is also uncertain. With the prominent position of the PaO_2_:FiO_2_ in the latest global definition of ARDS,^(1)^ the determination of optimum PaO_2_:FiO_2_ cut-off for prediction of mortality, and the associated discriminatory metrics need to be comprehensively evaluated to enable clinicians to make nuanced bedside decisions. Recent studies have highlighted the need for a paradigm shift in the management of ARDS, moving away from oxygenation-based indices for ventilator-induced lung injury, as these indices do not reflect differences in respiratory mechanics or ventilator requirements.^(11)^ Furthermore, PaO_2_:FiO_2_ is an unreliable metric at extremes of FiO_2_.^(12-14)^

To address this gap, we conducted this validation study to determine the optimal PaO_2_:FiO_2_ threshold and its discriminatory capacity for predicting mortality at various time points, including hospital mortality, 28-day, 90-day, 180-day, and 1-year mortality, in a large, prospectively collected, bi-national critical care dataset. Our objective was to determine the optimal PaO_2_:FiO_2_ threshold in the first 24 hours of ICU admission, and its associated discriminatory capacity, for prognostication of mortality among critically ill patients.

## METHODS

### Ethics

This study received approval with a waiver of consent from the Alfred Hospital Ethics Committee (Reference: 369/23). The Australian and New Zealand Intensive Care Society Centre for Outcome and Resource Evaluation (ANZICS CORE) Management Committee approved the use of the data from the ANZICS Adult Patient Database (APD) for this study. It has been reported in accordance with the Strengthening the Reporting of Observational Studies in Epidemiology (STROBE) guidelines.^(15)^

### Study design and participants

We conducted a retrospective cohort study using individual patient data from the ANZICS APD, which comprises 211 ICUs across Australia and New Zealand. We included all adults aged 16 years and over with an index admission to a participating ICU from January 1, 2018 through December 31, 2022 who had a documented arterial blood gas (ABG) within 24 hours of ICU admission. For patients with multiple ABGs within 24 hours, we used the ABG with the worst oxygenation score in the Acute Physiology and Chronic Health Evaluation (APACHE) III scoring system. Patients were excluded if they were transferred from another ICU, readmitted to the ICU during the same hospital admission, admitted solely for organ donation or palliative care, ABG data were missing, or missing data for hospital outcome.

### Data sources and definitions

The APD is a clinical quality registry collected and maintained by the ANZICS CORE that contains information on admissions to 98% of adult ICUs in Australia and 67% of ICUs in New Zealand.^(16)^ Data is collected using a standardised data dictionary (available on the ANZICS website, www.anzics.com.au) and data collectors undergo regular training. The data is scrutinised with regular quality assurance reviews with intermittent automated data checks.^(16)^ The database collects patient demographic details, chronic health parameters, and diagnostic, biochemical, and physiological information from the first 24 hours of ICU admission, as well as some interventions and outcomes at hospital discharge.

We extracted data on patient age, sex, comorbidities, ICU admission source, admission diagnosis, acute illness severity (using the APACHE II, APACHE III, Sequential Organ Failure Assessment [SOFA] scores and Australia New Zealand Risk of Death [ANZROD]), treatment limitations at admission to ICU, frailty status as measured by Clinical Frailty Scale (CFS), ICU organ supports, ICU and hospital mortality, ICU and hospital length of stay and discharge destinations (home and non-home discharge). Intensive care unit organ supports included invasive positive pressure ventilation (IPPV), noninvasive ventilation (NIV), renal replacement therapy, extracorporeal membrane oxygenation, and tracheostomy. We categorized patients using the oxygenation criterion of the New Global Definition of ARDS.^(1)^ Patients were categorised as having no AHRF (PaO_2_:FiO_2_ > 300), mild (PaO_2_:FiO_2_ 201 - 300), moderate (PaO_2_:FiO_2_ 101 - 200), or severe AHRF (PaO_2_:FiO_2_ ≤ 100). Post-discharge survival was obtained through linkages to the national death registries of Australia and New Zealand.

### Outcomes

The primary outcome was in-hospital mortality. Secondary outcomes included ICU mortality, mortality at 28 days, 90 days, 180 days, and 1 year, ICU and hospital lengths of stays, and discharge destination at hospital discharge. All patients who survived beyond one year were censored at this point.

### Statistical analysis

We summarized baseline ICU and patient-level characteristics and unadjusted outcomes using standard descriptive statistics. For categorical data, we used counts and percentages, and for continuous data, we used mean ± standard deviation (SD) or median (interquartile range [IQR]) as appropriate, depending on the distribution of data.

### Association between PaO2:FiO2 and hospital mortality

We evaluated the unadjusted association between PaO_2_:FiO_2_ and hospital mortality as a continuous, non-linear variable using restricted cubic splines with four knots. We calculated the specific unadjusted hospital mortality values for standard PaO_2_:FiO_2_ values by interrogating the spline curve.

### Validation of PaO2:FiO2 in predicting hospital mortality

To determine the optimal threshold for the PaO_2_:FiO_2_ in predicting hospital mortality, we generated receiver operating characteristic (ROC) curves. We then calculated the area under the curve (AUROC), and estimated 95% confidence intervals (95%CIs) around the AUROC using 1,000 bootstrap samples. A threshold level representing the highest sum of sensitivity and specificity based on each patient’s PaO_2_:FiO_2_ was calculated using the Youden method.^(17)^ In this method, we calculated the sensitivity and specificity over a range of PaO_2_:FiO_2_. For each value, we derived Youden’s J index using the following formula (Youden = sensitivity + specificity - 1). We identified the “optimal” PaO_2:_FiO_2_ threshold as the value that corresponds to the highest Youden index, reflecting the highest sum of sensitivity and specificity. We also calculated the negative predictive value (NPV) and positive predictive value (PPV) for this threshold. An additional sensitivity analysis was conducted, including only patients who met all the following criteria: were admitted to the ICU with a respiratory medical diagnosis, did not have any treatment limitations at ICU admission, and were in receipt of IPPV on Day 1 of ICU admission. These criteria were pre-specified to approximate a patient population that may be potentially eligible for inclusion in randomized trials investigating AHRF.

### Subgroup analyses

Patients were analysed for validation of the PaO_2_:FiO_2_ in prediction of hospital mortality in the following subgroups: those receiving invasive ventilation during the index ICU admission, receiving of other respiratory support (e.g., noninvasive ventilation, extracorporeal membrane oxygenation) during the ICU admission, sex, age categories (age less than 45 years, 45 - 64 years, 65 - 84 years, greater than 84 years), admission diagnoses (medical, cardiac surgery, neurosurgery/trauma, sepsis, post-operative), frailty category (not frail [CFS 1 - 3], mild frailty [CFS 4 - 5, moderate-severe frailty [CFS 6 - 8]) and the presence of treatment limitation status at ICU admission. The same methodology as above was employed. Comparison between subgroups (i.e., IPPV *versus* non-IPPV) was performed using DeLong’s method.^(18)^

Given the large number of patients in the dataset, a 2-sided p-value of 0.001 was used for statistical significance. Given the increased risk of type-1 error with multiple testing, the results of the secondary objectives should be viewed as exploratory. Hence, no adjustment for multiplicity was used. Only patients with complete data for all covariates were included in the analysis. Statistical analyses were performed using R Version 4.3.1 (R Core Team, R Foundation for Statistical Computing, Vienna, Austria) and RStudio Version 2023.12.1 (Posit Software, PBC, Boston, MA). Packages used for analysis included tidyverse, tidytable, and data.table, gtsummary, gt, cutpointr, and pROC. Reproducible code for the analysis is available online at https://github.com/BLMoran/AHRF_Validation.

### Role of the funding source

There was no funding source for this study.

## RESULTS

**Figure 1:**
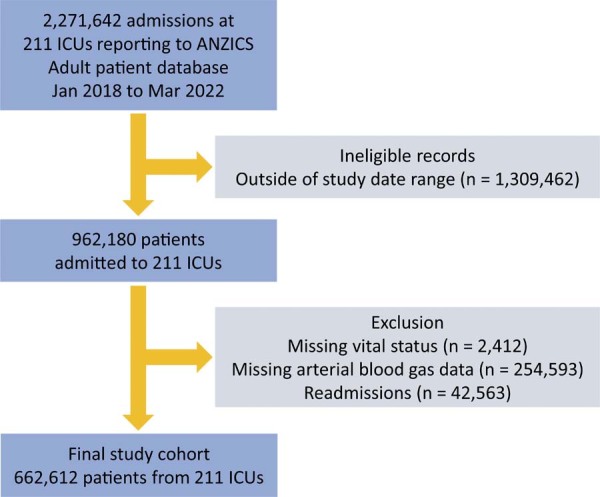
Patient recruitment flowchart.

During the study period, 662,612 patients met all inclusion and exclusion criteria (Figure 1). Acute hypoxaemic respiratory failure was present in 51% (337,811) of patients; 27% (181,499) of those were classified as mild AHRF, 19% (128,227) as moderate, and 4% (28,125) as severe. Acute hypoxaemic respiratory failure was not present in 49% (324,761) of patients. There were some differences in age and sex distribution between patients with AHRF and without AHRF, and between the AHRF categories, as shown in table 1. The APACHE II, APACHE III, ANZROD, and SOFA scores were all higher as AHRF severity increased. Distribution of other baseline characteristics are shown in table 1. The distribution of ICU interventions received by patients is shown in table 2.

ICU - intensive care unit; ANZICS - Australia New Zealand Intensive Care Society.

**Table 1:** Patient baseline characteristics

Characteristics	Overall	Acute hypoxaemic respiratory failure category			
		None (PFR > 300)	Mild (PFR 201 - 300)	Moderate (PFR 101 - 200)	Severe (PFR ≤ 100)
Number of patients	662,612	324,761	181,499	128,227	28,125
Age median (years)	66 (53 - 75)	65 (50 - 75)	68 (56 - 76)	67 (55 - 75)	65 (52 - 75)
Age category (years)					
< 44	99,985 (15)	60,314 (19)	19,751 (11)	15,515 (12)	4,405 (16)
> 84	47,408 (7.2)	24,404 (7.5)	13,341 (7.4)	7,985 (6.2)	1,678 (6.0)
45 - 64	198,888 (30)	94,398 (29)	54,892 (30)	40,546 (32)	9,052 (32)
65 - 84	315,855 (48)	145,422 (45)	93,376 (51)	64,083 (50)	12,974 (46)
Gender					
Female	275,502 (42)	146,046 (45)	70,722 (39)	48,420 (38)	10,314 (37)
Male	386,572 (58)	178,437 (55)	110,613 (61)	79,734 (62)	17,788 (63)
Intersex/indeterminate	378 (< 0.1)	200 (< 0.1)	108 (< 0.1)	52 (< 0.1)	18 (< 0.1)
Unknown	160 (< 0.1)	78 (< 0.1)	56 (< 0.1)	21 (< 0.1)	5 (< 0.1)
APACHE II Score	15 (11 - 20)	13 (10 - 18)	15 (12 - 20)	18 (14 - 24)	23 (18 - 30)
APACHE III Score	50 (38 - 66)	45 (34 - 59)	51 (39 - 66)	59 (46 - 77)	75 (57 - 99)
ANZROD	0.02 (0.01 - 0.07)	0.01 (0.00 - 0.04)	0.02 (0.01 - 0.07)	0.04 (0.01 - 0.17)	0.14 (0.03 - 0.45)
SOFA	4 (2 - 6)	3 (1 - 4)	4 (3 - 6)	5 (4 - 7)	7 (5 - 10)
Admission diagnosis					
Medical	197,553 (30)	74,842 (24)	51,037 (29)	55,029 (44)	16,645 (61)
Postoperative	191,807 (30)	112,503 (35)	53,720 (30)	22,809 (18)	2,775 (10)
Sepsis	52,894 (8.2)	23,625 (7.5)	14,518 (8.2)	11,587 (9.2)	3,164 (12)
Trauma/neurosurgery	77,311 (12)	50,410 (16)	18,202 (10)	7,660 (6.1)	1,039 (3.8)
Cardiac surgery	128,286 (20)	55,633 (18)	40,283 (23)	28,504 (23)	3,866 (14)
COVID-19 pneumonitis (Proven)	4,865 (0.7)	265 (<0.1)	657 (0.4)	2,569 (2.0)	1,374 (4.9)
Admission source					
Emergency department	158,625 (24)	67,604 (21)	41,263 (23)	38,897 (31)	10,861 (40)
Operating theatre/recovery	387,416 (59)	214,294 (67)	109,261 (61)	56,536 (45)	7,325 (27)
Ward	74,547 (11)	25,911 (8.1)	19,449 (11)	22,067 (18)	7,120 (26)
ICU, same hospital	658 (0.1)	276 (< 0.1)	168 (< 0.1)	165 (0.1)	49 (0.2)
Other hospital	29,930 (4.6)	12,646 (3.9)	8,065 (4.5)	7,396 (5.9)	1,823 (6.7)
Direct from home	675 (0.1)	298 (< 0.1)	172 (< 0.1)	158 (0.1)	47 (0.2)
Hospital type					
Tertiary	300,036 (45)	141,908 (44)	82,532 (45)	62,179 (48)	13,417 (48)
Metropolitan	94,431 (14)	39,233 (12)	25,487 (14)	22,858 (18)	6,853 (24)
Rural/regional	62,039 (9.4)	25,484 (7.8)	17,587 (9.7)	15,416 (12)	3,552 (13)
Private	206,106 (31)	118,136 (36)	55,893 (31)	27,774 (22)	4,303 (15)
Chronic respiratory disease	47,897 (7.2)	14,304 (4.4)	15,910 (8.8)	14,645 (11)	3,038 (11)
Chronic cardiovascular disease	60,583 (9.1)	26,453 (8.1)	17,915 (9.9)	13,388 (10)	2,827 (10)
Chronic hepatic disease	12,012 (1.8)	5,202 (1.6)	3,383 (1.9)	2,749 (2.1)	678 (2.4)
Chronic renal disease	23,002 (3.5)	10,724 (3.3)	6,478 (3.6)	4,720 (3.7)	1,080 (3.8)
Frailty					
Fit/well	279,572 (59)	147,891 (63)	73,133 (57)	48,604 (53)	9,944 (51)
Mild	142,545 (30)	64,841 (28)	41,025 (32)	30,223 (33)	6,456 (33)
Moderate/severe	50,901 (11)	20,270 (8.7)	15,069 (12)	12,546 (14)	3,016 (16)

PFR - PaO_2_:FiO_2_ ratio; APACHE - Acute Physiology and Chronic Health Evaluation; ANZROD - Australia New Zealand risk of death; SOFA - Sequential Organ Failure Assessment Score; ICU - intensive care unit. Results expressed as n, median (interquartile range), or n (%).

**Table 2:** Interventions in the intensive care unit

Characteristic	Overall	Acute respiratory failure category			
		None (PFR > 300)	Mild (PFR 201 - 300)	Moderate (PFR 101-200)	Severe (PFR < 100)
IPPV on Day 1	245,116 (37)	94,906 (29)	69,017 (38)	63,782 (50)	17,411 (62)
IPPV	254,129 (38)	97,466 (30)	71,640 (39)	66,789 (52)	18,234 (65)
Hours of IPPV (patients that received IPPV)	18 (8 - 62)	15 (7 - 41)	16 (7 - 49)	24 (10 - 89)	51 (16 - 156)
NIV	69,929 (11)	13,271 (4.1)	20,584 (11)	28,181 (22)	7,893 (28)
Hours of NIV (patients that received NIV)	13 (5 - 33)	9 (4 - 23)	13 (5 - 30)	15 (6 - 38)	15 (5 - 40)
ECMO	2,120 (0.3)	295 (< 0.1)	268 (0.1)	683 (0.5)	874 (3.1)
Inotropes/vasopressors	266,049 (40)	109,241 (34)	74,969 (41)	64,608 (50)	17,231 (61)
Tracheostomy	8,856 (1.3)	2,894 (0.9)	2,194 (1.2)	2,684 (2.1)	1,084 (3.9)
Acute renal failure*	30,315 (4.6)	10,503 (3.2)	7,892 (4.3)	8,427 (6.6)	3,493 (12)
Renal replacement therapy	27,885 (4.2)	8,775 (2.7)	6,738 (3.7)	8,515 (6.6)	3,857 (14)

PFR - PaO_2_:FiO_2_ ratio; IPPV - invasive positive pressure ventilation; NIV - noninvasive ventilation; ECMO - extracorporeal membrane oxygenation; * Acute renal failure is defined as urine output < 410mL in the first 24 hours in the intensive care unit AND serum creatinine > 133 micromol/L in a patient not receiving chronic dialysis. Results expressed as n (%) or median (interquartile range).

Overall, 8.6% (57,052) of patients died in the hospital. Hospital mortality was only 4.9% (15,797/324,761) among those without AHRF, while it was 12.2% (41,255/337,811) among those with AHRF. Among AHRF hospitals, mortality was 7.9% (14,291/181,499) for patients with mild AHRF, 14% (18,247/128,227) with moderate AHRF, and 31% (8,717/28,125) with severe AHRF (Table 3).

**Table 3:** Mortality outcomes

Characteristic	Overall	Acute hypoxaemic respiratory failure category			
		None (PFR > 300)	Mild (PFR 201 - 300)	Moderate (PFR 101 - 200)	Severe (PFR ≤ 100)
Number of patients	662,612	324,761	181,499	128,227	28,125
ICU mortality	38,681 (5.9)	9,138 (2.8)	8,897 (4.9)	13,212 (10)	7,434 (26)
Hospital mortality	57,052 (8.6)	15,797 (4.9)	14,291 (7.9)	18,247 (14)	8,717 (31)
28-day mortality	53,397 (8.1)	15,715 (4.8)	13,874 (7.6)	16,882 (13)	6,926 (25)
90-day mortality	70,842 (11)	23,570 (7.3)	18,689 (10)	20,734 (16)	7,849 (28)
180-day mortality	84,821 (13)	30,530 (9.4)	22,542 (12)	23,380 (18)	8,369 (30)
1-year mortality	104,441 (16)	40,498 (12)	27,923 (15)	27,008 (21)	9,012 (32)

PFR - PaO_2_:FiO_2_ ratio; ICU - intensive care unit. Results expressed as n or n (%).

**Figure 2:**
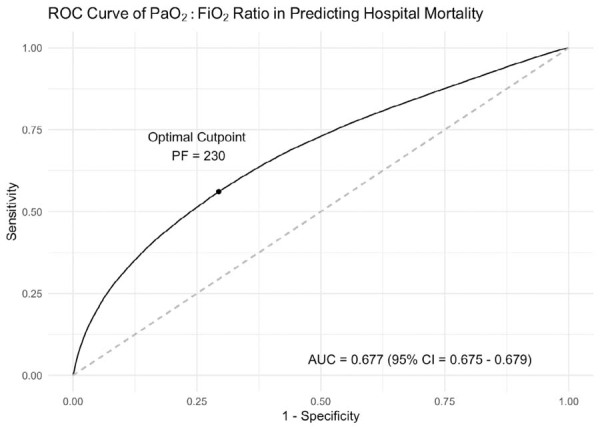
Receiver operating characteristic curve of PaO2:FiO2 for the prediction of hospital mortality.

The association between PaO_2_:FiO_2_ and hospital mortality was non-linear (Figure 1S - Supplementary Material) with an inflection point at a PaO_2_:FiO_2_ of approximately 200, below which the rise in hospital mortality with decreasing PaO_2_:FiO_2_ was steep. The AUROC (Figure 2) for PaO_2_:FiO_2_ in predicting the primary outcome was 0.677 (95%CI: 0.675 - 0.679). The optimal PaO_2_:FiO_2_ cut-point as determined by the Youden method was 230. The Youden’s index was 0.267 (Table 4). When using a PaO_2_:FiO_2_ of 230, there was a sensitivity of 56·1%, specificity of 70.6%, NPV of 94.5% and PPV of 15·2% for predicting hospital mortality. The calibration performance of the PaO_2_:FiO_2_ was excellent, as indicated by a low Brier score (0.0755), a calibration slope of 1.109, and a visual inspection of the calibration plot showing reasonable adherence to the diagonal (Figure 2S - Supplementary Material).

AUC - area under the curve; PF - PaO_2_:FiO_2_.

**Table 4:** Validation statistics

Criteria	Hospital mortality	ICU mortality	28-day mortality	90-day mortality	180-day mortality	1-year mortality
Optimal cut-point	230.000	224.286	233.333	233.333	233.333	233.333
Sensitivity	0.561	0.602	0.546	0.502	0.471	0.441
Specificity	0.706	0.719	0.694	0.696	0.696	0.697
Positive predictive value	0.152	0.118	0.135	0.165	0.186	0.214
Negative predictive value	0.945	0.967	0.946	0.921	0.900	0.869
Accuracy	0.694	0.712	0.682	0.675	0.668	0.656
Youden’s index	0.267	0.321	0.240	0.198	0.168	0.137
Area under the curve	0.677	0.709	0.659	0.632	0.612	0.591
Likelihood ratio positive	1.909	2.144	1.785	1.651	1.552	1.453
Likelihood ratio negative	0.622	0.554	0.654	0.716	0.759	0.803

ICU - intensive care unit.

When including only patients with a respiratory medical diagnosis, no treatment limitations at ICU admission, and IPPV requirement on Day 1 (14,356/662,612, 2.2%), the shape of the curve was similar to the entire cohort; however, the inflection point was reduced to 130 (Figure 3S - Supplementary Material). The AUROC (Figure 4S - Supplementary Material) for PaO_2_:FiO_2_ in predicting hospital mortality was 0.591 (95%CI: 0.583 - 0.606).

Secondary outcomes are presented in tables 3 and 1S (Supplementary Material). Overall mortality increased from 5.9% (38,681/662,612) at ICU discharge to 8.1% (53,397/662,612) at 28 days, and to 16% (104,441/662,612) at 1 year, with an expected increase in mortality at each time interval as the severity of AHRF increased. The AUROCs for the PaO_2_:FiO_2_ in predicting mortality at ICU discharge, 28 days, 90 days, 180 days, and 1 year were similar and ranged from 0.591 to 0.709 (Figures 5S to 9S - Supplementary Material). There was a slight progressive decrease in the AUROC from 28-day to 1-year mortality. The optimal PaO_2_:FiO_2_ cut-points were similar and ranged from 224 to 233. The Youden indices, sensitivities, specificities, NPVs, and PPVs for all secondary outcomes were similar to those for hospital mortality (Table 4).

A range of prespecified subgroups were examined, with AUROC comparisons and curves presented in table 2S and figures 10S - 18S (Supplementary Material). While statistical significance was reached in numerous subgroup comparisons, no subgroup showed an AUROC for the PaO_2_:FiO_2_ in predicting hospital mortality that improved by a clinically significant magnitude. The AUROCs in the subgroup analyses ranged from 0.516 (NIV) to 0.704 (age less than 44 years).

## DISCUSSION

In this retrospective study examining 662,612 patients across 211 ICUs in Australia and New Zealand over a 5-year period, we found that the optimal PaO_2_:FiO_2_ for prognosticating hospital mortality was 230. However, the discriminatory capacity of the PaO_2_:FiO_2_ in predicting hospital mortality was low (AUROC = 0.677), with correspondingly low sensitivity and specificity. There were no clinically significant improvements in these metrics when mortality at other time-points, including ICU discharge, 28 days, 90 days, 180 days, and 1 year, were evaluated, nor were there any subgroups where PaO_2_:FiO_2_ performed better at predicting hospital mortality, even though it remained a predictor of mortality up to 1 year after ICU admission.

A 13,408-patient retrospective study from Toronto, Canada, investigating the association between mechanical ventilation intensity and mortality, demonstrated a non-linear relationship between the PaO_2_:FiO_2_ and ICU mortality, with an inflection point at approximately 200.^(19)^ Another retrospective study with 2,729 patients had similar findings, with an inflection point for the relationship between PaO_2_:FiO_2_ and a composite outcome of mortality or ventilator dependence at ICU discharge, detected at 200.^(20)^ Both studies were performed for purposes other than determining the validity of the PaO_2_:FiO_2_ in predicting mortality, and neither included critically ill patients not requiring mechanical ventilation. Our much larger study confirms that the optimal cut-point for PaO_2_:FiO_2_ in predicting mortality is approximately 200. Significantly, our study adds to the literature by showing that the predictive capability of the PaO_2_:FiO_2_ for mortality at various time points is minimal, and it should be used very cautiously for this purpose.

Our findings suggest that the PaO_2_:FiO_2_ measured in the first 24 hours of ICU admission alone has a minimal role in prognostication for critically ill patients requiring ICU admission. It has low discriminatory capacity, sensitivity, and specificity. Nonetheless, the NPV for the PaO_2_:FiO_2_ was high, and thus, in specific settings, a “normal” PaO_2_:FiO_2_ may be reassuring when predicting survival. However, for overall prognostication, it may be better to use more comprehensive scoring or illness severity evaluation systems, such as SOFA, APACHE-II, APACHE-III, ANZROD, or similar. These tools capture a much broader range of acute derangements beyond hypoxaemia and, beyond SOFA, also incorporate severe chronic comorbidities. Thus, although the PaO_2_:FiO_2_ is simple, easy, and quick to measure at the bedside, it may be more accurate and meaningful to use other, more comprehensive tools. The major limitation of these tools is that they require large amounts of data and are complex to calculate at the bedside. While these tools may be helpful for research, they are not well-suited for clinical use. An incremental approach, which starts with the PaO_2_:FiO_2_ and sequentially adds readily available bedside data to generate a predictive tool with higher discriminatory capacity, sensitivity, and specificity, may be helpful to clinicians.

A subset of patients with AHRF will meet criteria for ARDS. The oxygenation indices play a prominent role in the 2023 Global Definition of ARDS,^(1)^ with a PaO_2_:FiO_2_ of less than 300 required for diagnosis of ARDS. Given the limited prognostic performance of the PaO_2_:FiO_2_ for mortality at different time points, we question its reliability for predicting outcomes. This challenges the use of ARDS definitions for prognostic enrichment, a common practice in clinical trials, and of management strategies based on PaO_2_:FiO_2_ thresholds. Furthermore, the diagnosis of ARDS in non-intubated patients requires only a PaO_2_:FiO_2_ < 300, without further categorisation into mild, moderate, or severe, as for intubated patients. Since the relationship between the PaO_2_:FiO_2_ and mortality is non-linear, with a steep increase only when the ratio drops below approximately 200, the absence of sub-categorization for non-intubated patients into mild, moderate, and severe ARDS may lead to misclassifying a significant proportion of low-risk patients as having ARDS. Our findings add weight to recent arguments about the limitations of PaO_2_:FiO_2_ as a defining criterion for ARDS.^(11)^

Our results also suggest that the AHRF severity categories, defined by arbitrary PaO_2_:FiO_2_ thresholds, may need to be redefined using data-driven methods. The current definitions, used as they are for prognostication and clinical trials enrolment, could be improved upon to better enable them to serve their intended purposes. For example, it may be more clinically relevant to diagnose AHRF at the PaO_2_:FiO_2_ inflection point of 200, with potential further sub-categorisations at lower PaO_2_:FiO_2_. Furthermore, for patients who have a respiratory diagnosis and require mechanical ventilation on day 1 of ICU admission, a group that more closely mimics a clinical trial population than the whole registry cohort, the inflection point was even lower at a PaO_2_:FiO_2_ of 130, making the arbitrary categories of mild and moderate ARDS even less relevant.

The nature of the dataset limits this study. The ANZICS APD is a benchmarking database, with data collected primarily for quality assurance purposes. Research is a secondary use of the database that it was not explicitly designed for. Only a single PaO_2_:FiO_2_ is available from the worst ABG analysis within the first 24 hours of ICU admission. This may not reflect the patients’ condition beyond the first 24 hours, and it may exclude patients who subsequently develop AHRF after 24 hours in ICU. It may also over-categorise patients who recover quickly,^(21)^ for example with acute cardiogenic pulmonary oedema, as having severe AHRF. Granular data on ICU interventions and therapies were not available. While ANZICS APD coverage of Australian ICUs is near complete, it covers only approximately 67% of ICUs in New Zealand. Findings from Australia and New Zealand ICUs may not be generalisable globally, particularly in low- and middle-income countries with less well-resourced healthcare systems. While Youden’s index is an established method for determining optimal cut-offs, it has some limitations: it does not account for the clinical consequences of false positives and false negatives (which vary widely depending on the clinical condition), nor does it consider disease prevalence. Thus, the real-world applicability of the cut-off determined by Youden’s index may be limited.

However, it does have several strengths, including a large, multicentre, binational dataset linked to long-term survival outcomes. The primary findings were consistent across multiple patient types including both intubated and non-intubated patients. The findings were also clinically plausible and robust in sensitivity analyses and within the prespecified subgroups.

## CONCLUSION

The PaO_2_:FiO_2_ has limited discriminatory capacity for the prognostication of mortality among critically ill patients with acute hypoxaemic respiratory failure, including those who require mechanical ventilation for respiratory diagnoses. The optimal PaO_2_:FiO_2_ threshold for mortality prognostication was 230. However, our findings suggest that the additional value of the PaO_2_:FiO_2_ in current prognostic scoring systems and entry into clinical trials should be reconsidered.

## SUPPLEMENTARY MATERIAL

Supplementary material 1

## References

[1] Matthay MA, Arabi Y, Arroliga AC, Bernard G, Bersten AD, Brochard LJ (2024). A New Global Definition of Acute Respiratory Distress Syndrome. Am J Respir Crit Care Med.

[2] Villar J, Mora-Ordoñez JM, Soler JA, Mosteiro F, Vidal A, Ambrós A (2022). The PANDORA Study: Prevalence and Outcome of Acute Hypoxemic Respiratory Failure in the Pre-COVID-19 Era. Crit Care Explor.

[3] Villar J, Blanco J, Añón JM, Santos-Bouza A, Blanch L, Ambrós A, ALIEN Network (2011). The ALIEN study: incidence and outcome of acute respiratory distress syndrome in the era of lung protective ventilation. Intensive Care Med.

[4] Brun-Buisson C, Minelli C, Bertolini G, Brazzi L, Pimentel J, Lewandowski K, ALIVE Study Group (2004). Epidemiology and outcome of acute lung injury in European intensive care units. Results from the ALIVE study. Intensive Care Med.

[5] Bellani G, Laffey JG, Pham T, Fan E, Brochard L, Esteban A, LUNG SAFE Investigators, ESICM Trials Group (2016). Epidemiology, patterns of care, and mortality for patients with acute respiratory distress syndrome in intensive care units in 50 countries. JAMA.

[6] Luhr OR, Antonsen K, Karlsson M, Aardal S, Thorsteinsson A, Frostell CG, The ARF Study Group (1999). Incidence and mortality after acute respiratory failure and acute respiratory distress syndrome in Sweden, Denmark, and Iceland. Am J Respir Crit Care Med.

[7] Herridge MS, Cheung AM, Tansey CM, Matte-Martyn A, Diaz-Granados N, Al-Saidi F, Canadian Critical Care Trials Group (2003). One-year outcomes in survivors of the acute respiratory distress syndrome. N Engl J Med.

[8] Palakshappa JA, Krall JT, Belfield LT, Files DC (2021). Long-term outcomes in acute respiratory distress syndrome: epidemiology, mechanisms, and patient evaluation. Crit Care Clin.

[9] Azoulay E, Vincent JL, Angus DC, Arabi YM, Brochard L, Brett SJ (2017). Recovery after critical illness: putting the puzzle together-a consensus of 29. Crit Care.

[10] Ling RR, Ponnapa Reddy M, Subramaniam A, Moran B, Ramanathan K, Ramanan M (2024). Epidemiology of acute hypoxaemic respiratory failure in Australian and New Zealand intensive care units during 2005-2022. A binational, registry-based study. Intensive Care Med.

[11] Catozzi G, Pozzi T, Nocera D, Donati B, Giovanazzi S, Ghidoni V (2025). Rethinking ARDS classification: oxygenation impairment fails to predict VILI risk. Intensive Care Med.

[12] Nirmalan M, Willard T, Columb MO, Nightingale P (2001). Effect of changes in arterial-mixed venous oxygen content difference (C(a-v)O2) on indices of pulmonary oxygen transfer in a model ARDS lung. Br J Anaesth.

[13] Hahn CE (2001). KISS and indices of pulmonary oxygen transfer. Br J Anaesth.

[14] Karbing DS, Kjaergaard S, Smith BW, Espersen K, Allerød C, Andreassen S (2007). Variation in the PaO2/FiO2 ratio with FiO2: mathematical and experimental description, and clinical relevance. Crit Care.

[15] von Elm E, Altman DG, Egger M, Pocock SJ, Gøtzsche PC, Vandenbroucke JP, STROBE Initiative (2007). The Strengthening the Reporting of Observational Studies in Epidemiology (STROBE) statement: guidelines for reporting observational studies. Lancet.

[16] Australian and New Zealand Intensive Care Society (ANZICS) (2020). Centre for Outcome and Resource Evaluation 2020 Report.

[17] Youden WJ (1950). Index for rating diagnostic tests. Cancer.

[18] DeLong ER, DeLong DM, Clarke-Pearson DL (1988). Comparing the areas under two or more correlated receiver operating characteristic curves: a nonparametric approach. Biometrics.

[19] Urner M, Jüni P, Hansen B, Wettstein MS, Ferguson ND, Fan E (2020). Time-varying intensity of mechanical ventilation and mortality in patients with acute respiratory failure: a registry-based, prospective cohort study. Lancet Respir Med.

[20] Ruan SY, Huang CT, Chang HT, Liu WL, Wang WJ, Tseng YT (2022). Construct validity of PaO _2_/FiO _2_ ratios in defining acute respiratory distress syndrome. Am J Respir Crit Care Med.

[21] Schenck EJ, Oromendia C, Torres LK, Berlin DA, Choi AM, Siempos II (2019). Rapidly Improving ARDS in Therapeutic Randomized Controlled Trials. Chest.

