# Supplementary material for: Validation of PaO2:FiO2 for predicting hospital mortality in critically ill patients with acute hypoxaemic respiratory failure: a retrospective binational registry-based study
Source: Crit Care Sci. 2026 Feb 26;38:e20260221. doi: 10.62675/2965-2774.20260221 (PMC13124106; doi:10.62675/2965-2774.20260221)
Supplement: Supplementary material 1 [file 2965-2774-ccsci-38-e20260221-suppl01.pdf]

Critical Care Science
https://doi.org/10.62675/2965-2774.20260221

Validation of PaO_2:FiO_2 for predicting hospital mortality in critically ill patients with acute hypoxaemic respiratory failure: a retrospective binational registry-based study

Mahesh Ramanan[1], Benjamin Moran[2], Ryan Ruiyang Ling[3], Aidan Burrell[4], Ashwin Subramaniam[5], Kollengode Ramanathan[6], Mallikarjuna Ponnappa Reddy[7], David Pilcher[8], Kiran Shekar[9] on behalf of the Australasian Acute Hypoxaemic Respiratory Failure Investigators (AAHRFI)

Table 1S - Patient outcomes

Characteristic	Overall	Acute respiratory failure category			
		None (PFR> 300)	Mild (PFR 200 - 300)	Moderate (PFR 100-200)	Severe (PFR < 100)
Hospital mortality	57,052 (8.6)	15,797 (4.9)	14,291 (7.9)	18,247 (14)	8,717 (31)
ICU mortality	38,681 (5.9)	9,138 (2.8)	8,897 (4.9)	13,212 (10)	7,434 (26)
ICU length of stay	3.33 (5.57)	2.60 (4.19)	3.23 (5.32)	4.64 (7.13)	6.35 (9.50)
ICU length of stay	1.89 (0.96 - 3.70	1.63 (0.90 - 2.88)	1.92 (0.98 - 3.67)	2.79 (1.46 - 5.15)	3.48 (1.54 -7 .43)
Hospital length of stay)	15 (86)	14 (87)	15 (84)	16 (88)	17 (76)
Hospital length of stay	9 (5 - 15)	8 (4 - 14)	9 (5 - 16)	10 (6 - 17)	10 (4 - 19)

PFR - PaO_2:FiO_2 ratio; ICU - intensive care unit. Results expressed as n (%), mean (standard deviation), or median (interquartile range).

Table 2S - Subgroup comparisons

Comparison	AUC 1 versus AUC 2	p value
Day 1 IPPV		
Yes *versus* No	0.628 *versus* 0.701	< 0.001
IPPV any time		
Yes *versus* No	0.627 *versus* 0.698	< 0.001
NIV any time		
Yes *versus* No	0.622 *versus* 0.668	< 0.001
ECMO any time		
Yes *versus* No	0.516 *versus* 0.675	< 0.001
Cardiac *versus* neurosurgery	0.62 *versus* 0.641	0.008
Cardiac *versus* postoperative	0.62 *versus* 0.647	< 0.001
Cardiac *versus* medical	0.62 *versus* 0.644	< 0.001
Cardiac *versus* sepsis	0.62 *versus* 0.661	< 0.001
Neurosurgery *versus* postoperative	0.641 *versus* 0.647	0.326
Admission diagnosis		
Neurosurgery *versus* medical	0.641 *versus* 0.644	0.587

Continue...

This is an open access article under the CC BY license (https://creativecommons.org/licenses/by/4.0/).

...continuation

Neurosurgery *versus* sepsis	0.641 *versus* 0.661	0.001
Postoperative *versus* medical	0.647 *versus* 0.644	0.422
Postoperative *versus* sepsis	0.647 *versus* 0.661	0.006
Medical *versus* sepsis	0.644 *versus* 0.661	< 0.001
APACHE 3 score		
> 50 *versus* ≤ 50	0.626 *versus* 0.613	0.01
Age categories		
Age < 44 *versus* age 45 - 64	0.704 *versus* 0.675	< 0.001
Age < 44 *versus* age 65 - 84	0.704 *versus* 0.67	< 0.001
Age < 44 *versus* age > 84	0.704 *versus* 0.67	< 0.001
Age 45 - 64 *versus* age 65 - 84	0.675 *versus* 0.67	0.116
Age 45 - 64 *versus* age > 84	0.675 *versus* 0.67	0.309
Age 65 - 84 *versus* age > 84	0.67 *versus* 0.67	0.96
Sex		
Male *versus* female	0.671 *versus* 0.684	< 0.001
Frailty		
Fit/Well *versus* mild frailty	0.687 *versus* 0.667	< 0.001
Fit/well *versus* moderate/severe frailty	0.687 *versus* 0.638	< 0.001
Mild frailty *versus* moderate/severe frailty	0.667 *versus* 0.638	< 0.001
Treatment limitations		
Yes *versus* no	0.624 *versus* 0.680	< 0.001

AUC - area under curve; IPPV - invasive positive pressure ventilation; NIV - noninvasive ventilation ECMO - extracorporeal membrane oxygenation; APACHE - Acute Physiology and Chronic Health Evaluation.

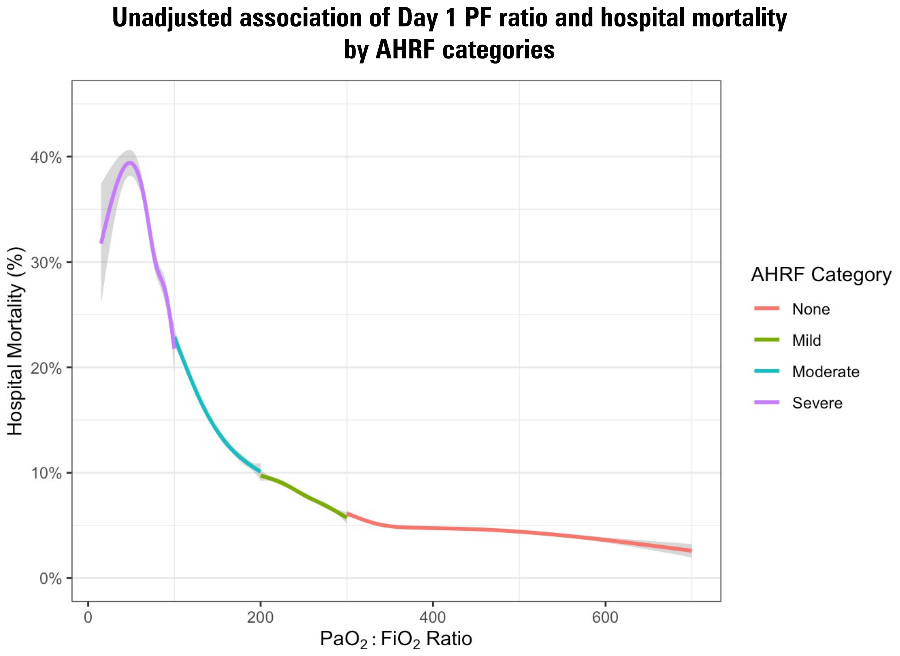

PF ratio - PaO_2:FiO_2 ratio; AHRF - acute hypoxaemic respiratory failure.

Figure 1S - Hospital mortality by acute hypoxaemic respiratory failure category.

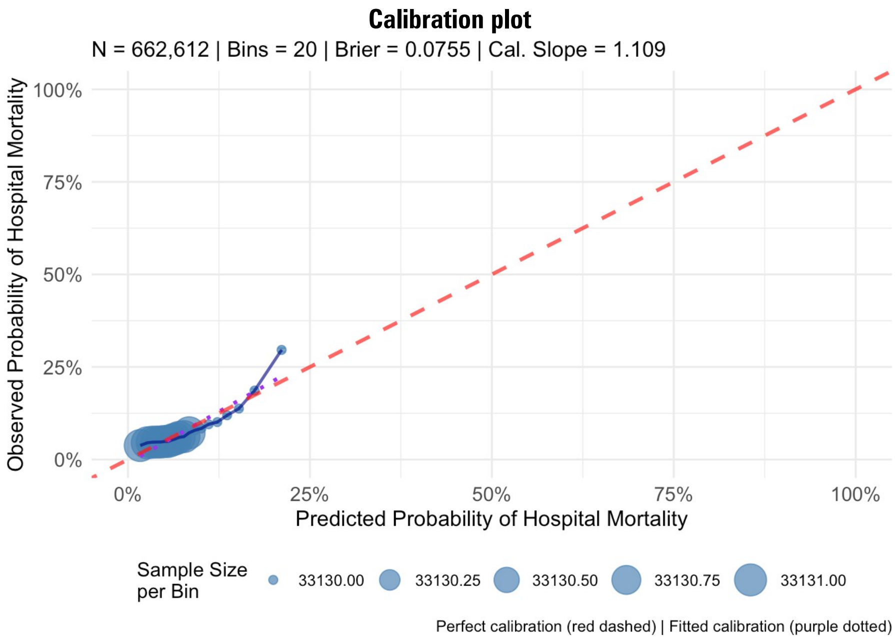

Metric	Value	Interpretation
Brier score	0.0755	Lower better (0 = perfect)
Calibration slope	1.1090	Should ≈ 1.0
Mean calibration error	0.0161	Lower better
Max calibration error	0.0850	Lower better

Figure 2S - Calibration.

The calibration performance of PaO_2:FiO_2 ratio was excellent, as the Brier score was low (0.0755), the calibration slope was close to 1.0 (1.109), and visual inspection of the calibration plot showed reasonable adherence to the diagonal. However, the discrimination performance was modest. The area under the receiver operator curve showed fair discrimination but was not strong. The Youden's index was 0.267, indicating moderate optimal performance at the cutoff value. Hence, the PaO_2:FiO_2 ratio has good calibration with modest discrimination. This is interpreted such that the PaO_2:FiO_2 ratio can predict hospital mortality accurately (i.e. good calibration) but has limited ability to separate high-risk from low-risk patients (i.e., modest discrimination). This is reflected in the likelihood ratios, where both the positive and negative test provide only a slight increase or decrease in probability, and is in the "minimal clinical impact" range.

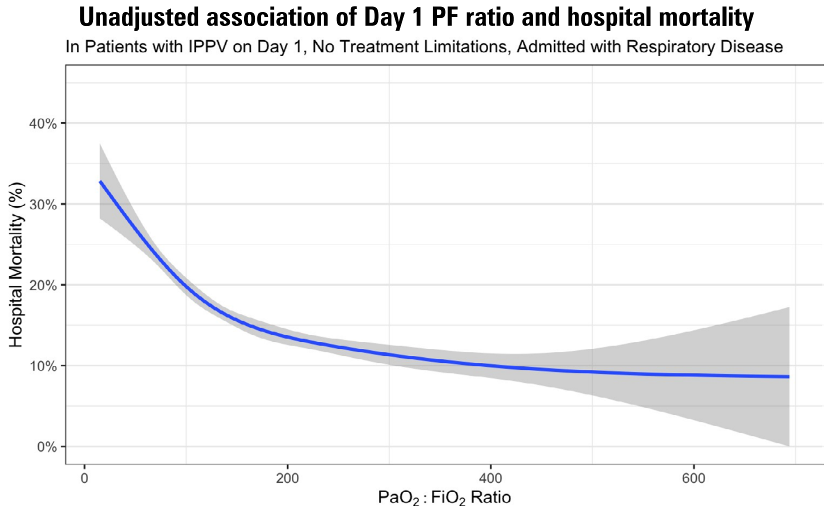

PF ratio - PaO_2:FiO_2 ratio; IPPV - invasive positive pressure ventilation.

Figure 3S - Unadjusted association between day PaO_2:FiO_2 ratio and hospital mortality in patients requiring invasive ventilation on Day 1 of intensive care unit admission with a respiratory diagnosis and no treatment limitations.

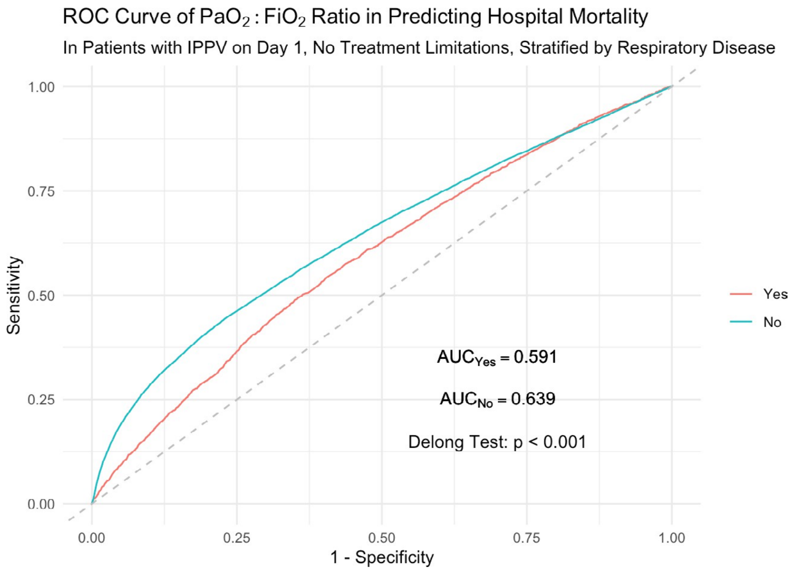

ROC - receiver operating characteristic; IPPV - invasive positive pressure ventilation AUC - area under the curve.

Figure 4S - Receiver operating characteristic curve of PaO_2:FiO_2 ratio in predicting hospital mortality: patients receiving invasive ventilation of Day 1 of intensive care unit admission with respiratory diagnosis and no treatment limitations.

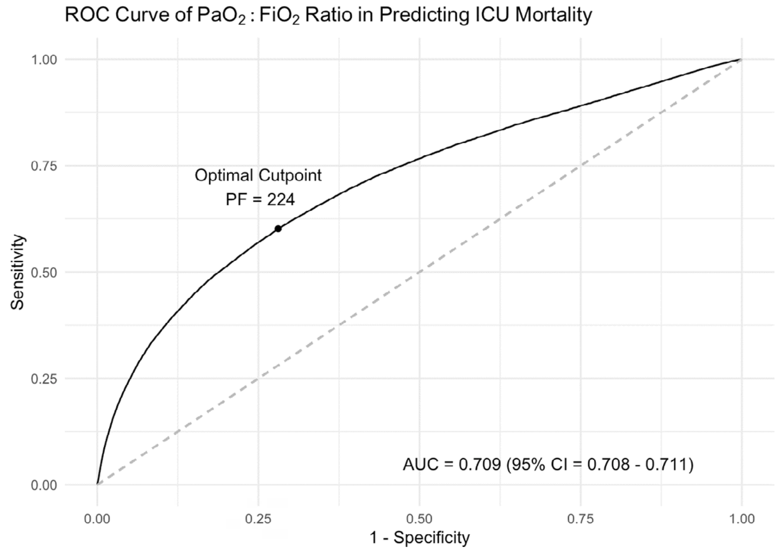

ROC - receiver operating characteristic; ICU - intensive care unit; AUC - area under the curve.

Figure 5S - Receiver operating characteristic curve of PaO_2:FiO_2 ratio in predicting intensive care unit mortality.

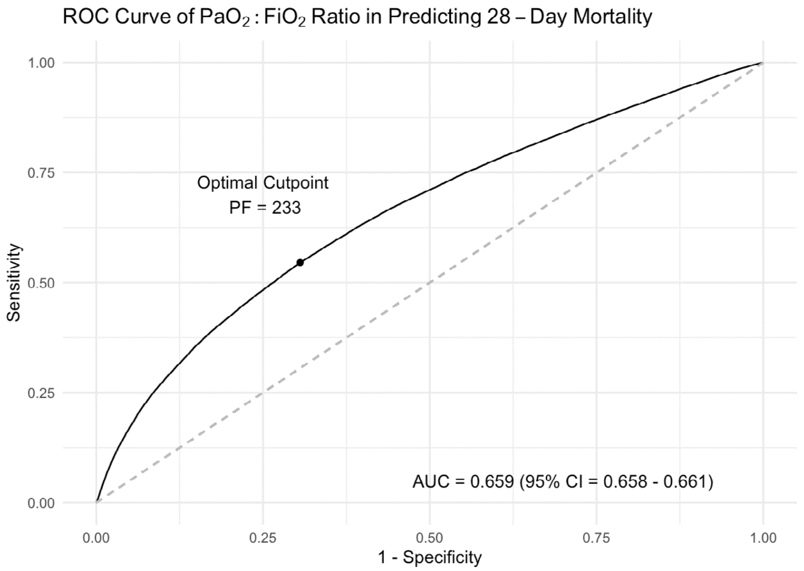

ROC - receiver operating characteristic; AUC - area under the curve.

Figure 6S - Receiver operating characteristic curve of PaO_2:FiO_2 ratio in predicting Day 28 mortality.

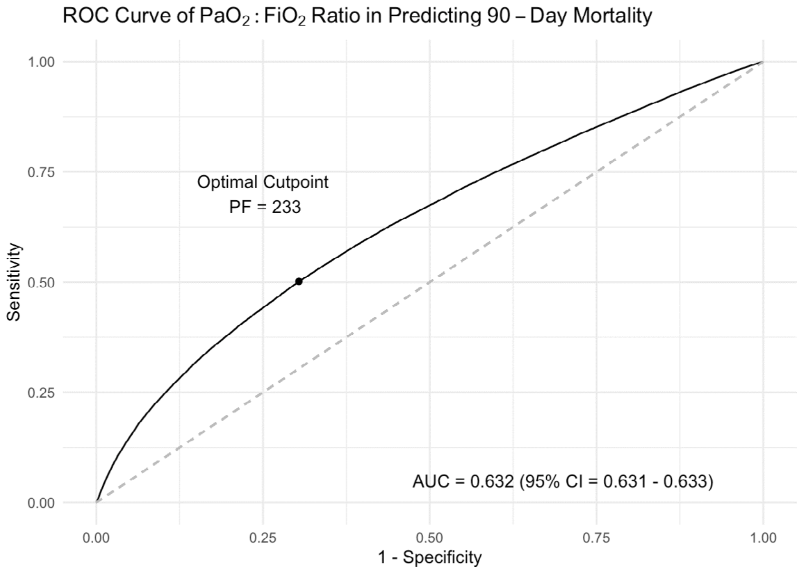

ROC - receiver operating characteristic; AUC - area under the curve.

Figure 7S - Receiver operating characteristic curve of PaO_2:FiO_2 ratio in predicting Day 90 mortality,

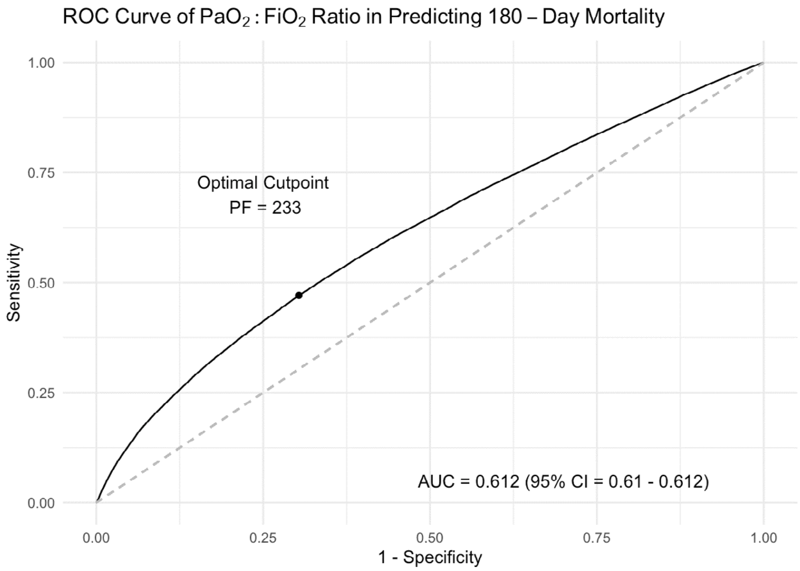

ROC - receiver operating characteristic; AUC - area under the curve.

Figure 8S - Receiver operating characteristic curve of PaO_2:FiO_2 ratio in predicting Day 180 mortality.

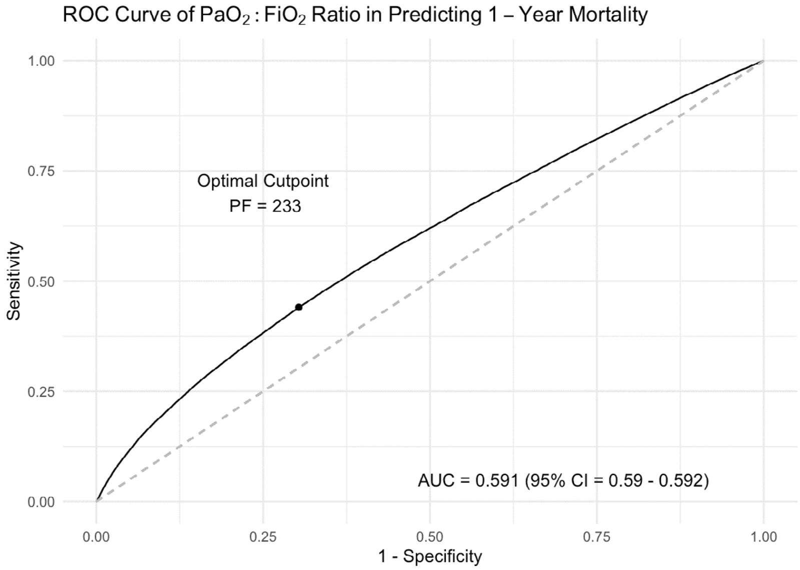

ROC - receiver operating characteristic; AUC - area under the curve.

Figure 9S - Receiver operating characteristic curve of PaO_2:FiO_2 ratio in predicting 1-year mortality.

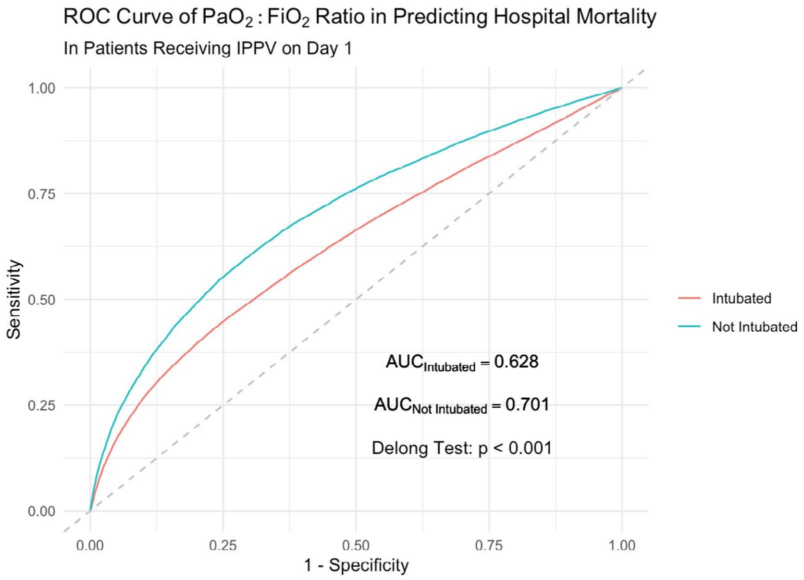

ROC - receiver operating characteristic; IPPV - invasive positive pressure ventilation; AUC - area under the curve.

Figure 10S - Receiver operating characteristic curve of PaO_2:FiO_2 ratio in predicting hospital mortality by day 1 intubation status.

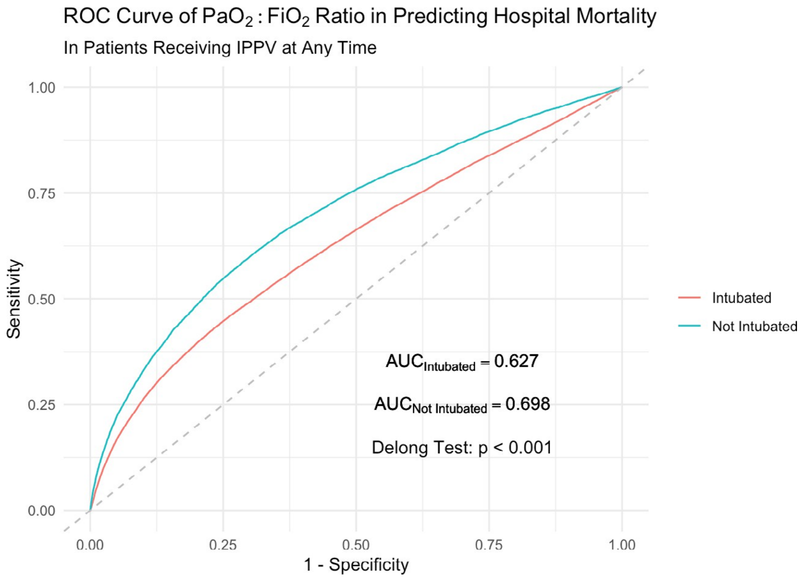

ROC - receiver operating characteristic; IPPV - invasive positive pressure ventilation; AUC - area under the curve.

Figure 11S - Receiver operating characteristic curve of PaO_2:FiO_2 ratio in predicting hospital mortality by intubation status.

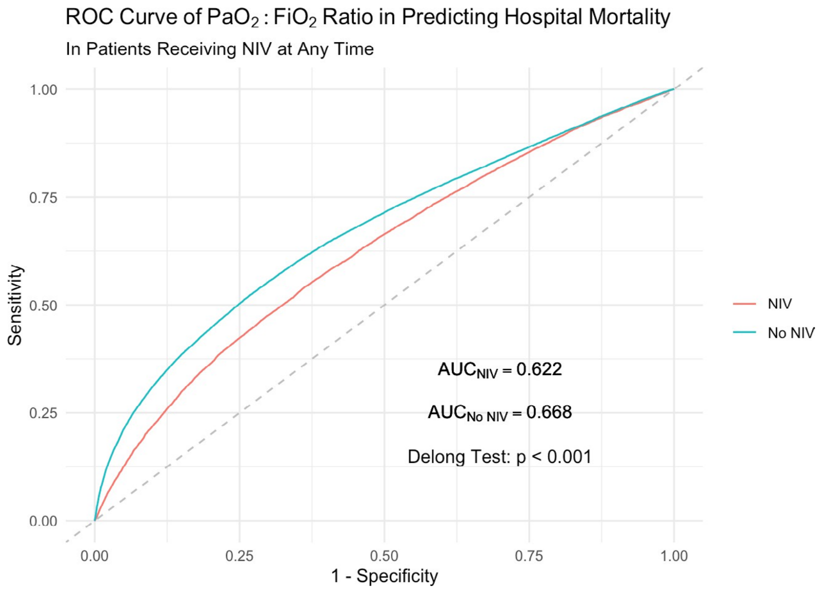

ROC - receiver operating characteristic; NIV – noninvasive ventilation; AUC - area under the curve.

Figure 12S - Receiver operating characteristic curve of PaO_2:FiO_2 ratio in predicting hospital mortality by requirement for non-invasive ventilation.

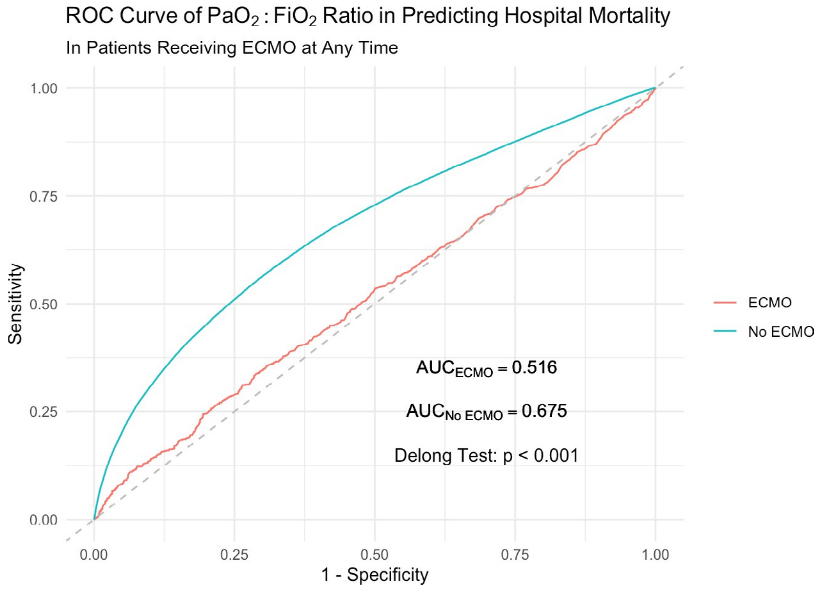

ROC - receiver operating characteristic; ECMO - extracorporeal membrane oxygenation; AUC - area under the curve.

Figure 13S - Receiver operating characteristic curve of PaO_2:FiO_2 ratio in predicting hospital mortality by requirement for extracorporeal membrane oxygenation.

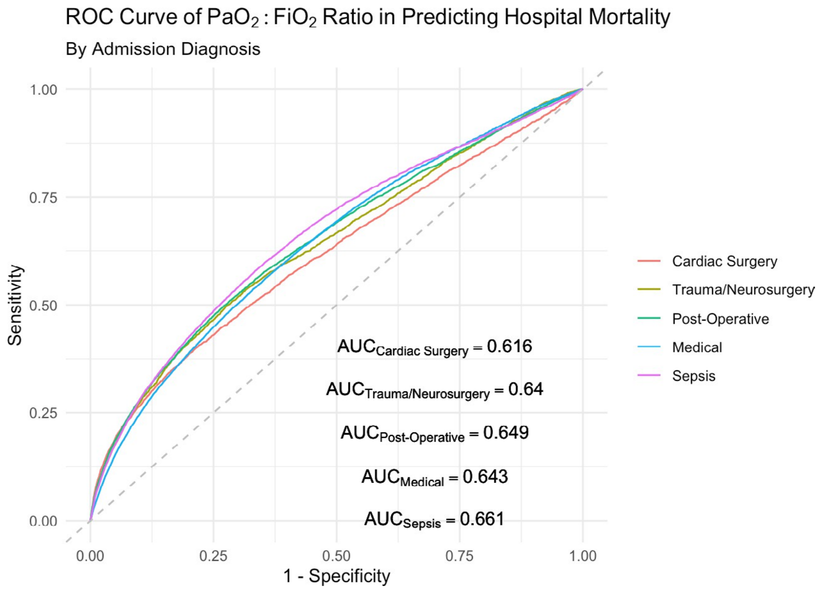

ROC - receiver operating characteristic; AUC - area under the curve.

Figure 14S - Receiver operating characteristic curve of PaO_2:FiO_2 ratio in predicting hospital mortality by admission diagnostic category.

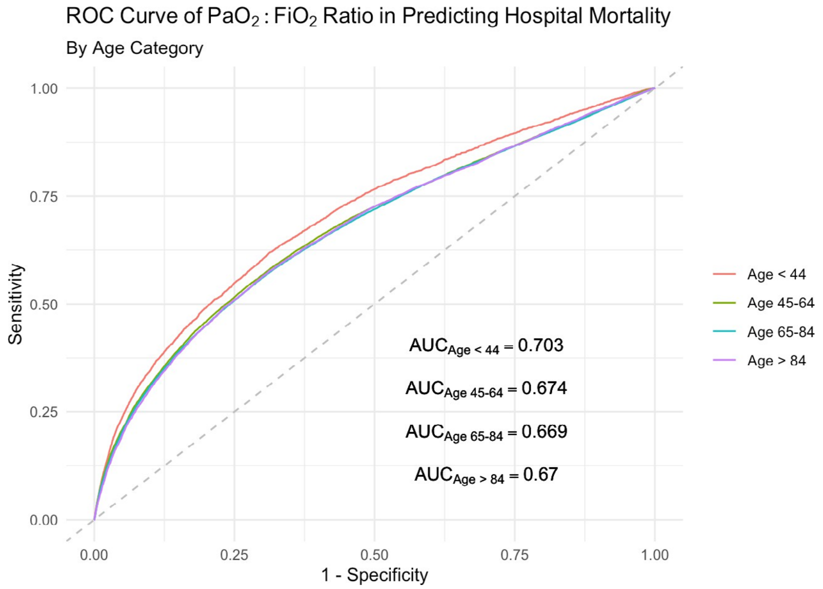

ROC - receiver operating characteristic; AUC - area under the curve.

Figure 15S - Receiver operating characteristic curve of PaO_2:FiO_2 ratio in predicting hospital mortality by age group.

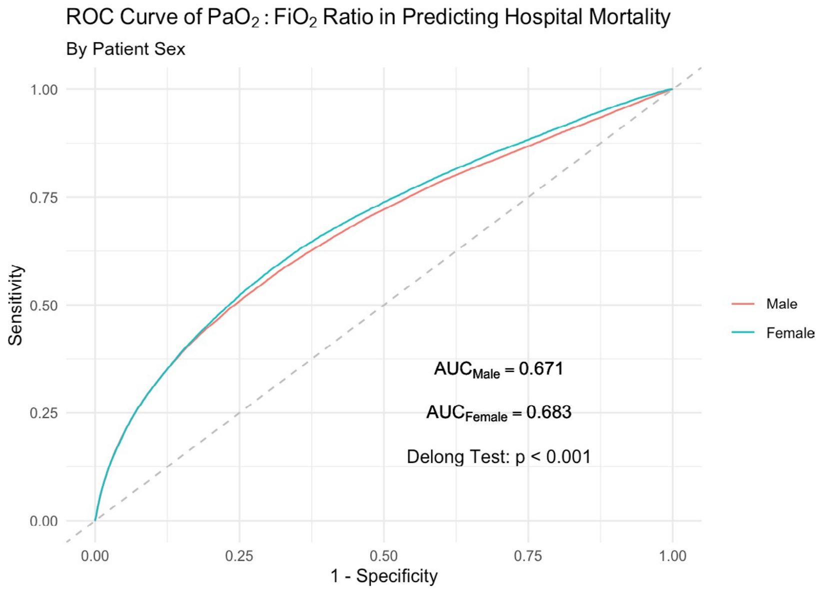

ROC - receiver operating characteristic; AUC - area under the curve.

Figure 16S - Receiver operating characteristic curve of PaO_2:FiO_2 ratio in predicting hospital mortality by sex.

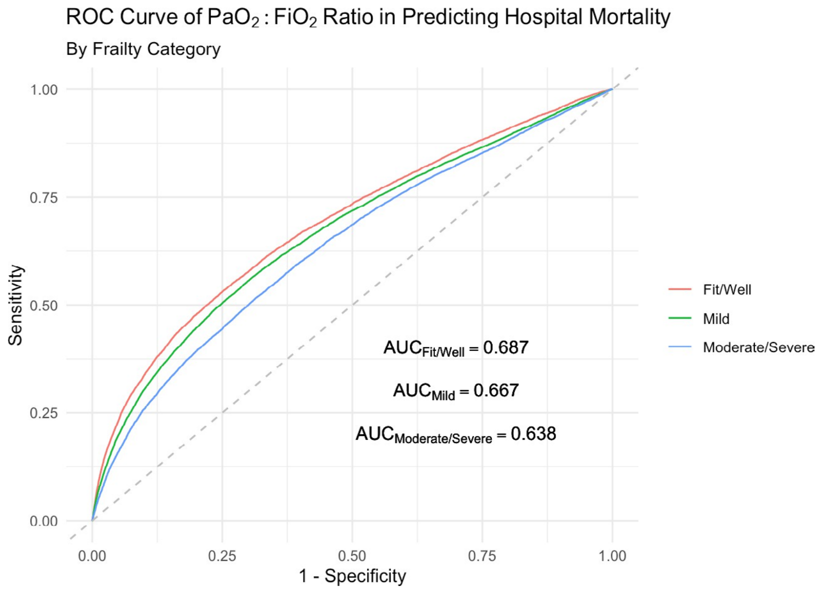

ROC - receiver operating characteristic; AUC - area under the curve.

Figure 17S - Receiver operating characteristic curve of PaO_2:FiO_2 ratio in predicting hospital mortality by frailty category.

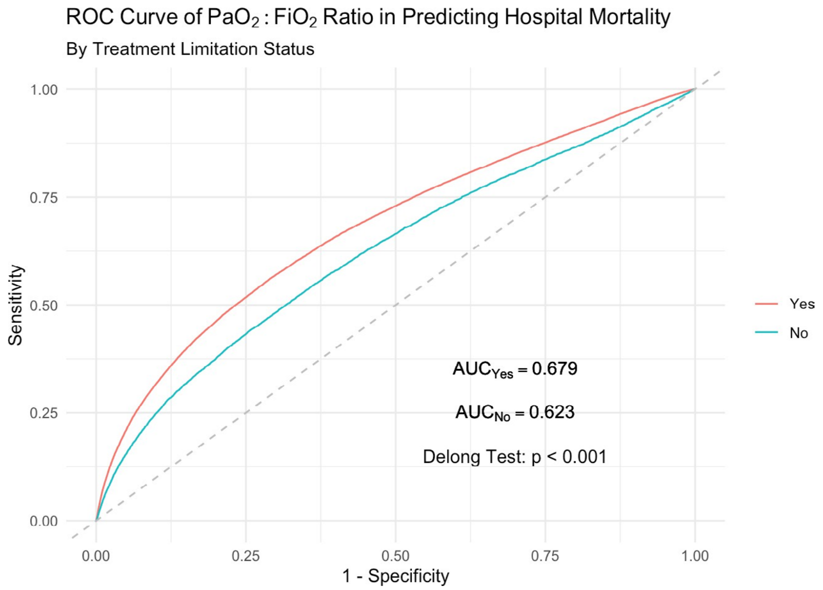

ROC - receiver operating characteristic; AUC - area under the curve.

Figure 18S - Receiver operating characteristic curve of PaO_2:FiO_2 ratio in predicting hospital mortality by treatment limitations status.

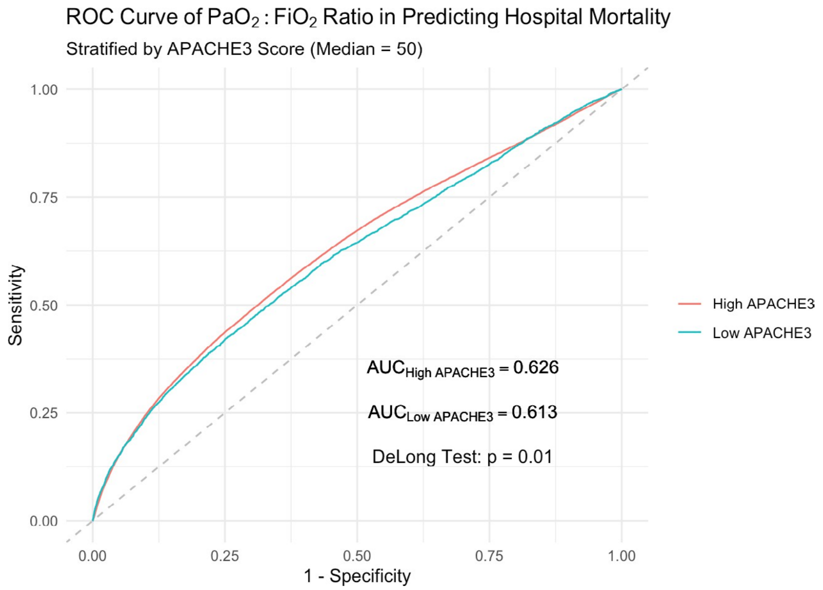

ROC - receiver operating characteristic; APACHE - Acute Physiology and Chronic Health; AUC - area under the curve.

Figure 19S - Receiver operating characteristic curve of PaO_2:FiO_2 ratio in predicting hospital mortality by APACHE-3 score.
